# Multi-user frequency selective beam steering by reconfigurable intelligent surfaces in the Ka-band

**DOI:** 10.1038/s41598-025-95063-1

**Published:** 2025-03-29

**Authors:** Lukas Mueller, Alexander Wolff, Steffen Klingel, Janis Krieger, Lars Franke, Ralf Stemler, Marco Rahm

**Affiliations:** Department of Electrical and Computer Engineering and Research Center OPTIMAS, RPTU Kaiserslautern-Landau, Kaiserslautern, D-67663 Germany

**Keywords:** RIS, Beam Steering, Multi User, Dual Frequency, Machine Learning, Electrical and electronic engineering, Electronic and spintronic devices, Information theory and computation

## Abstract

Reconfigurable intelligent surfaces (RIS) have been proposed to extend the coverage of wireless communication signals at mm-wave frequencies. Here, we designed and fabricated a reconfigurable intelligent surface (RIS) for multi-user frequency selective beam steering (MU-FSBS) that achieves higher channel capacity than traditional time division multiple access (TDMA) techniques in multi-user communication scenarios. MU-FSBS is enabled by a varactor diode-tuned RIS that allows to steer several beams in the Ka-band independently at the same time. For such targeted multi-frequency beam steering, we implemented a customized neural network-based machine learning architecture specifically designed to optimize the bias voltage patterns of the RIS. As an experimental demonstration, we first accomplished independent beam steering of normally incident beams at 27 GHz and 31 GHz to deflection angles between 10$^\circ$ and 45$^\circ$ in a defined plane. Secondly, we compared the achievable channel capacities of the proposed MU-FSBS approach with those of TDMA, finding an average increase of approximately 50% in channel capacity at a fixed SNR of 20 dB. At an SNR of 60 dB, MU-FSBS even demonstrated a remarkable 84% increase in channel capacity compared to TDMA.

## Introduction

**Fig. 1:**
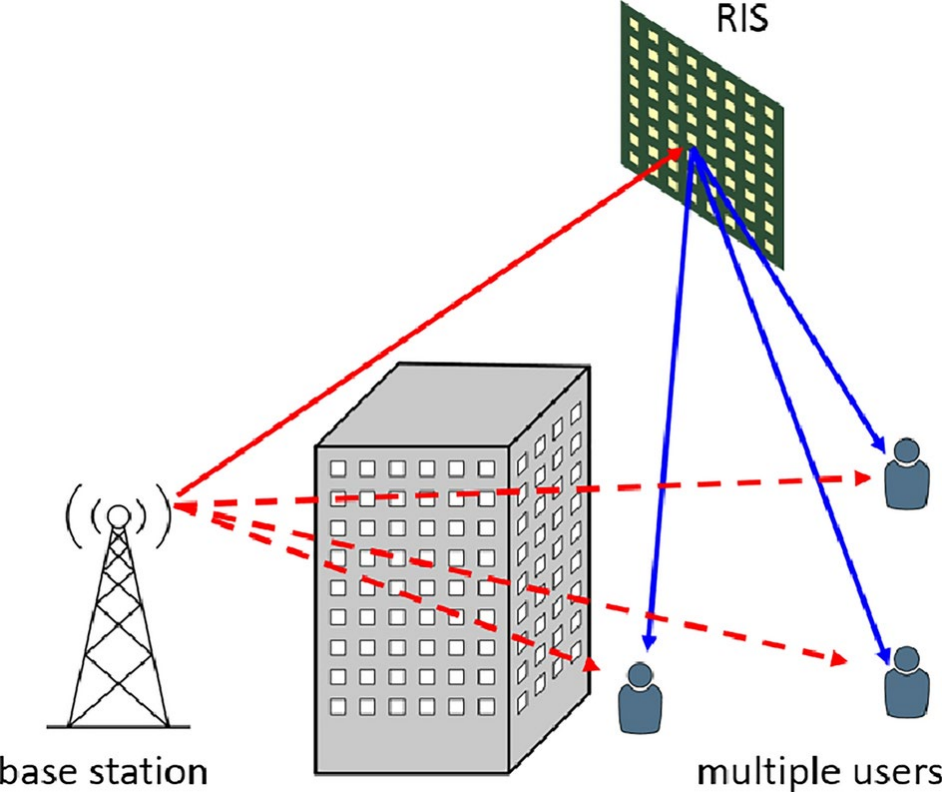
Use case for a RIS. The direct lines of sight between the base station and the users are obstructed. The RIS can provide virtual lines of sight to each user.

In 5G and 6G wireless communication, the obstruction of beams by buildings or other obstacles is a more severe problem than in earlier generations of mobile communication due to the use of radiation in higher frequency bands^1^. In face of this challenge, Reconfigurable Intelligent Surfaces (RIS), i.e. reconfigurable metasurfaces, have been proposed as a possible solution for redirecting radiation from a base station towards obstructed areas as sketched in Fig. 1. In order to maximize the available signal power at the locations of the users, the most commonly discussed operation mode of the RIS is the beam forming of a single incident beam into a deflected single or into deflected multiple pencil beams as depicted in Fig. 1^2–11^.

In this operation mode, multiple users can be served by a multiplexed signal, as used for example in time division multiple access (TDMA), orthogonal frequency division multiple access (OFDMA) or beam division multiple access (BDMA). While time slicing in TDMA allows multiple users to share the same frequency band at different time slots at the cost of time resources, BDMA is independent of frequency, time or code restrictions as the beam of a base station is divided into multiple beams that serve multiple users at different locations without interference. In OFDMA again, information is transmitted at multiple carrier frequencies. Nevertheless, OFDMA, as heavily used in 4G cellular networks, remains a relevant multiplexing technique even in the 5G or 6G environment. For this reason, the use of a RIS can offer a number of advantages as they enable frequency-selective, independent and smartly tunable deflection of an incident beam into multiple pencil beams that serve multiple users at different locations. In our specific case, the RIS is used to demultiplex a normally incident two-carrier frequency signal into two distinct pencil beams, which makes it operate as a space division demultiplexer as it obviously translates frequency information into space information.

Previous research has proposed static^12,13^ and reconfigurable metasurface designs^14–19^ to be used as RIS in more than one frequency band. However, no such designs have been proposed for wireless communication applications in the Ka-band, where specific frequency ranges are reserved for the 5G NR standard^20^, by now.

In this work, we capitalize on the dispersion of the varactor diode-based RIS from our previous work^2^ to create two independently steerable pencil beams at 27 GHz and 31 GHz with the same bias voltage pattern. The RIS hardware is presented in detail in section "RIS-hardware" and our previous publications^2,21^. The bias voltage-dependence of the RIS’s scattering parameters forces strict boundary conditions on the optimization of the bias voltage pattern and renders it non-trivial. In order to find suitable bias voltage matrices for simultaneous, but independent two-color beam steering, we cannot apply techniques described in previous literature because they are either only designed for single frequency multi-beam steering^22–24^ or limited to binary phase values^25,26^. We therefore combined neural network techniques with Kirchhoff diffraction theory in a semi-analytical approach to calculate the optimal bias voltages for the RIS. For the sake of simplicity, we shortly refer to this method as Multi User-Frequency Selective Beam Steering (MU-FSBS) in the remainder of this publication.

For verification of the proposed method, we experimentally demonstrate simultaneous beam steering of two beams at 27 GHz and 31 GHz within a single plane to deflection angles between $10^\circ$ and $45^\circ$. By comparing our proposed MU-FSBS method with TDMA, we find that MU-FSBS has the potential to yield higher data rates than TDMA above a critical signal to noise ratio.

## Multi user-frequency selective beam steering (MU-FSBS)

Figure 1 illustrates a typical use case for a RIS. A base station radiates a wireless communication signal towards a RIS from where it is distributed towards multiple users via beam forming. The scope of our beam steering method MU-FSBS is to increase the achievable total transmitted data rate over the RIS. In MU-FSBS, we simultaneously provide separate users with signals of different carrier frequencies via a single RIS. However, choosing the right bias voltage pattern for this application is not trivial. Our method for obtaining such a pattern will be presented in section "Optimization framework". When optimizing a bias voltage pattern for the RIS on several carrier frequencies simultaneously, finding a compromise for the signal quality of each user is unavoidable. For simplicity, we limited the evaluation in this work to two users although, in general, more users could be addressed by MU-FSBS, yet complicating the optimization problem. The supplementary material to this publication contains a generalized description of the MU-FSBS algorithm for more than two users.

In a thorough study, we evaluate the benefit of MU-FSBS with respect to channel capacity *C*, which describes the theoretical upper limit of the achievable data rate in a communication channel without error in the presence of noise^27^. The Shannon-Hartley theorem describes the channel capacity *C* as follows:

1 $$\begin{aligned} C = B \log _2\left( 1+S/N\right) \end{aligned}$$

where *B* is the bandwidth of the channel, *S* is the average signal power over the bandwidth at the receiver and *N* is the noise power level at the receiver over the bandwidth. The ratio *S*/*N* is called the signal to noise ratio (SNR). As the total channel capacity in MU-FSBS $C_\text {MU}$ we consider the sum of the channel capacities for the separate carrier frequencies as shown in Eq. (2). Here, we assume that the noise power level *N* is identical for both users and that the available bandwidth *B* around both carrier frequencies is identical. The signal power at each user’s location is indicated with the superscripts (1) and (2), respectively.

2 $$\begin{aligned} C_\text {MU} = B \left( \log _2\left( 1+S_\text {MU}^{(1)}/N\right) + \log _2\left( 1+S_\text {MU}^{(2)}/N\right) \right) \end{aligned}$$

For comparison, we also evaluate the channel capacity $C_\text {TD}$ for TDMA (time division multiple access) with a channel bandwidth *B* that is equal to the channel bandwidth *B* we assumed in each frequency band of MU-FSBS. In TDMA, the available reception time slots are divided between the users. Under the assumption that both users are allocated the same total reception time, the total channel capacity for TDMA $C_\text {TD}$ is the mean value of the channel capacities of the two users as shown in Eq. (3).

3 $$\begin{aligned} C_\text {TD} = \frac{1}{2} B\left( \log _2\left( 1+S_\text {TD}^{(1)}/N\right) + \log _2\left( 1+S_\text {TD}^{(2)}/N\right) \right) \end{aligned}$$

Note that the total occupied bandwidth for the MU-FSBS method in this example is twice as large as the occupied bandwidth in TDMA, if we assume the bandwidth *B* around each carrier frequency to be identical to the bandwidth *B* in TDMA.

MU-FSBS requires to simultaneously steer two beams at two frequencies to the different positions of the two considered user areas. Due to this compromise, the received signal powers of the individual users tend to be lower in MU-FSBS than in TDMA. Using the total transmitted power $S_0$ as a reference, we can express the total received power for MU-FSBS and TDMA as $S_\text {MU} = \left| {h_\text {MU}} \right| ^2 S_0$ and $S_\text {TD} = \left| {h_\text {TD}} \right| ^2 S_0$, respectively. Here, $\left| {h_\text {MU}} \right| ^2$ and $\left| {h_\text {TD}} \right| ^2$ are the path losses. Given a fixed noise power level *N*, we can calculate a minimum required $S_0 = S_\text {crit}$ and consequently a critical $\text {SNR}_\text {crit}$, for which $C_\text {MU}\ge C_\text {TD}$.

4 $$\begin{aligned} \begin{aligned} \text {SNR}_\text {crit}&= \frac{S_\text {crit}}{N} \\ \text {SNR}_\text {crit} [\hbox {dB}]&= S_\text {crit} [\text {dBm}] - {N} [\text {dBm}] \end{aligned} \end{aligned}$$

**Fig. 2:**
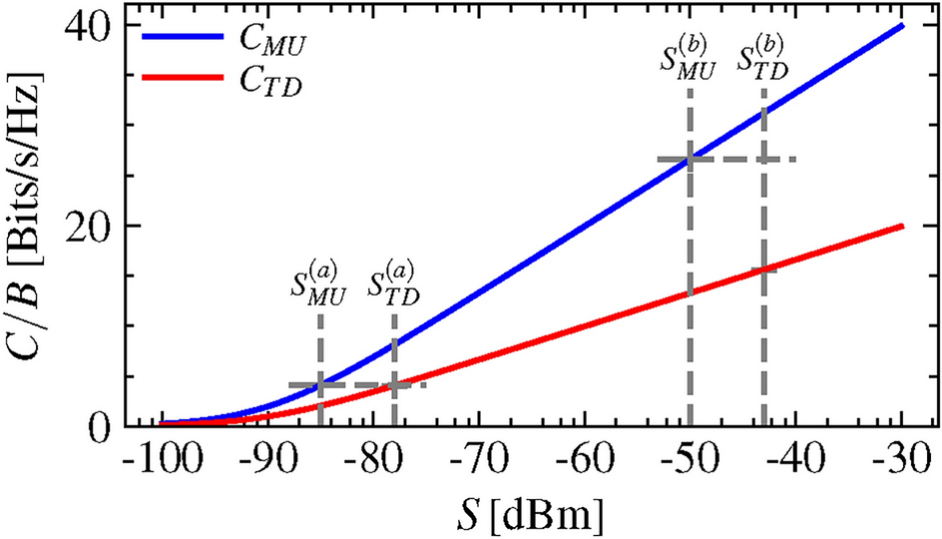
Channel capacity of MU-FSBS vs. TDMA. Influence of received signal power on channel capacity per bandwidth according to Eq. (2) for MU-FSBS and Eq. (3) for TDMA. A noise power level of $N=-90\,\text {dBm}$ and the two exemplary transmitted signal powers $S_0^{(a)}=-75\,\text {dBm}$ and $S_0^{(b)}=-40\,\text {dBm}$ as well as the path losses $S_{MU} = 0.1 S_0$ and $S_{TD} = 0.5 S_0$ are assumed. The critical SNR of 15 dB for this example is reached at $-75\,\text {dBm}$.

We illustrate this fact with the following exemplary configuration. In this example, we presume a noise power level of $N = -90\,\text {dBm}$ and fixed path losses of $\left| {h_{MU}} \right| ^2 = 0.1$ and $\left| {h_{TD}} \right| ^2 = 0.5$. Additionally, we assume that both users receive the same power in each example case. Under these conditions, the bandwidth-normalized channel capacities $C_{MU}/B$ and $C_{TD}/B$ look as illustrated in Fig. 2, when plotted versus the total received signal powers $S_\text {MU}$ and $S_\text {TD}$, respectively. Both curves are monotonously increasing and we find that for $S_0 = -75\,\text {dBm}$ (case (a)) $C_{MU}$ and $C_{TD}$ are equal. For higher values of $S_0$, i.e. $-40\,\text {dBm}$ (case (b)), $C_{MU} > C_{TD}$. Therefore, the critical SNR in this example is $\text {SNR}_\text {crit} = -75\,\text {dBm} - (-90\,\text {dBm}) = {15}\, \hbox {dB}$ according to Eq. (4). In section "Beam steering results and verification" the critical SNR will be used as the distinctive parameter to assess the benefit of MU-FSBS over TDMA.

## RIS-hardware

**Fig. 3:**
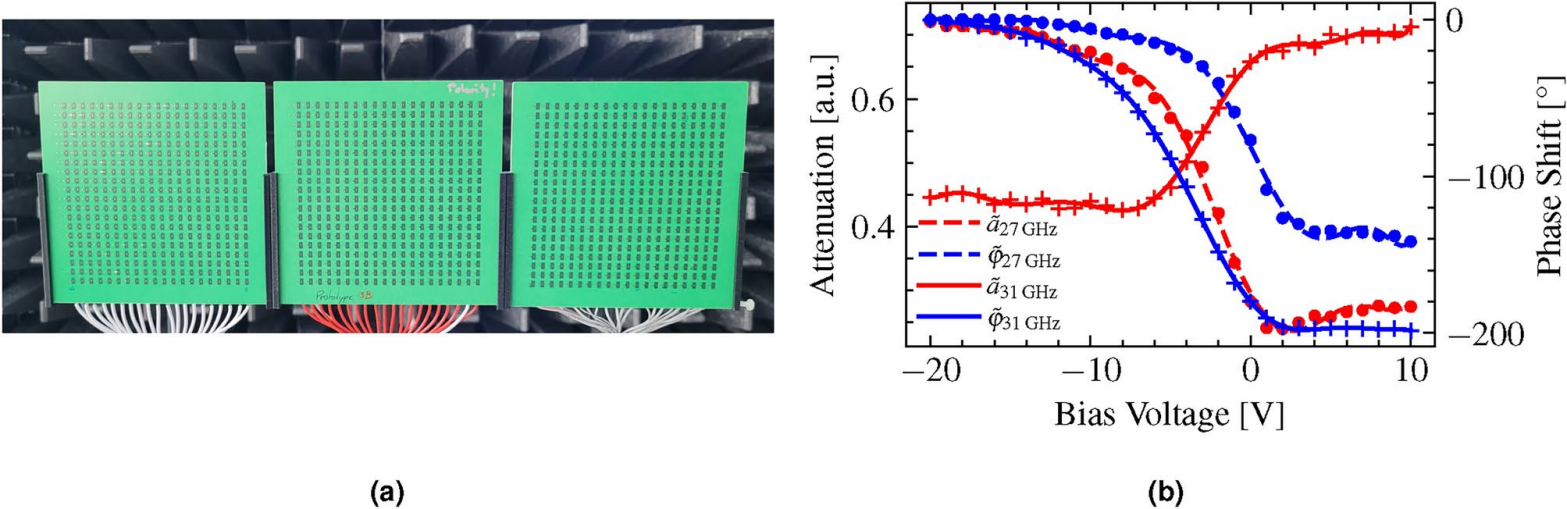
RIS hardware and characterization. (**a**) Front side of the multi-modular RIS. Microwave absorbers are located in the background. (**b**) Attenuation *a* and phase shift $\varphi$ of the RIS unit cell for ${27}\, \hbox {GHz}$ (circles) and ${31}\, \hbox {GHz}$ (crosses) at the measured bias voltages. The lines indicate 10^th^ order polynomial fits $\tilde{a}$ for the attenuation and $\tilde{\varphi }$ for the phase shift of the RIS unit cell.

The RIS used in this work comprises three identical modules aligned in a row, each measuring 128 mm x 128 mm. Every module consists of 20 x 20 square cut unit cells with a width of 5.33 mm and a 10.66 mm wide unstructured perimeter around the edges. The module and unit cell configuration have been detailed in prior research^2^. The three assembled modules are interspersed by two 5.3 mm wide gaps, which results in the overall dimensions of the RIS of 128 mm x 395 mm. Figure 3a visualizes the front side of the multi-modular RIS. Each unit cell contains a varactor diode, whose impedance can be individually tuned by bias voltage. As a result, we can locally control the phase of the waves reflected on each unit cell. This allows us to shape the diffracted beam by application of specific bias voltage patterns across the RIS unit cells. Note that in our particular configuration, all 20 unit cells per column of the RIS are vertically interconnected, which confines our ability to adjust the phase only in a column-wise manner, thus limiting beam-steering to a single plane. To characterize the RIS, we measured the dependence of the complex $S_{31}$-parameter of the RIS on the bias voltage $V_B$ that was uniformly applied to all unit cells as described in our earlier work^2^. Note, that we placed the receiving antenna (port 3) in the maximum of the 0^th^ order of backwards diffraction for the measurement. We normalized the magnitude $\text {mag}(S_{31})$ to its maximum value for each frequency, separately, which yields the attenuation $a_{27\,\text {GHz}}(V_B)$ and $a_{31\,\text {GHz}}(V_B)$ of the RIS unit cell. Furthermore, we referenced the phase of the $S_{31}$-parameter by subtracting its value at $V_B = -20V$ for each frequency, separately, to get the phase shifts $\varphi _{27\,\text {GHz}}(V_B)$ and $\varphi _{31\,\text {GHz}}(V_B)$ of the RIS unit cell. The crosses and circles in Fig. 3b show the attenuation and phase shift of the RIS unit cell for 27 GHz and 31 GHz at the measured bias voltages. Subsequently, we fitted these attenuation and phase shift values with 10^th^ order polynomials $\tilde{a}(V_B)$ and $\tilde{\varphi }(V_B)$ for both carrier frequencies. The polynomials are illustrated as solid and dashed lines in Fig. 3b. From the fitted curves $\tilde{a}(V_B)$ and $\tilde{\varphi }(V_B)$ we can calculate the continuous scattering parameter $\tilde{S}_{31}(V_B)$ as follows:

5 $$\begin{aligned} \tilde{S}_{31}(V_B) = \tilde{a}(V_B) \exp \left\{ j\frac{\pi }{180}\tilde{\varphi }(V_B)\right\} \end{aligned}$$

We apply this bias voltage-dependent continuous representation of the $\tilde{S}_{31}$-parameter during the training process of our machine learning architecture, the details of which we present in section "Optimization framework".

## Optimization framework

The proposed MU-FSBS requires dynamic steering of two beams at different carrier frequencies to spatially separate, moving positions $P_I$ and $P_{II}$. To achieve this, we developed an optimization framework for calculating the required bias voltages that must be applied to each individual unit cell of the RIS. Hereby, we directly exploit the frequency and bias voltage dependent phase shift and attenuation of the reflected partial waves from each individual unit cell in Eq. (5) to accurately calculate the spatial electric field distribution of the reflected waves from the RIS in the observation plane by use of Kirchhoff diffraction theory. The subsequent optimization process is then based on a comparison of the calculated field distribution with the desired target distributions in both frequency bands. The remainder of this section is structured as follows: first we describe the Kirchhoff computation of the back-diffracted electric fields before embedding it in a machine learning architecture for optimization of the bias voltage patterns.

### Field evaluation using Kirchhoff diffraction theory

**Fig. 4:**
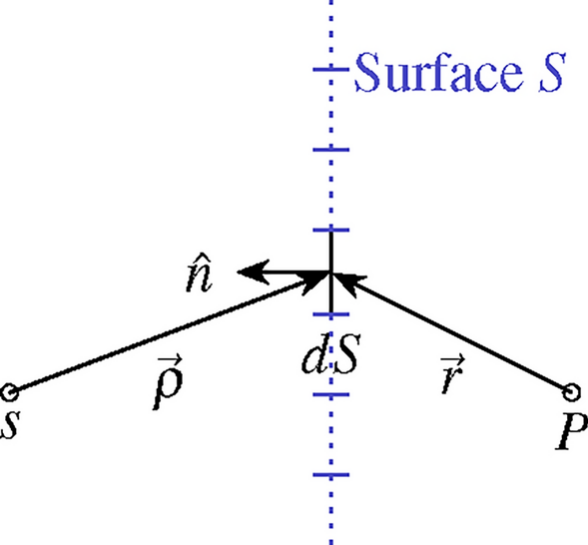
Schematic of the quantities used in the Kirchhoff diffraction formula: A wave emitted from source *s* passes through the surface *S* with the complex aperture function $f_S$ and results in the electric field $\mathscr {E}_P$ at point *P*.

The field evaluation method presented in this section is based on the Kirchhoff diffraction formula, which computes the electric field $\mathscr {E}_P$ of a wave with wave number *k*, that was emitted by a source at position *s*, at the spatial position *P* (see Fig. 4)^28,29^

6 $$\begin{aligned} \mathscr {E}_P = \int _S f_S \frac{\exp \left\{ jk(\rho +r)\right\} }{\rho r} \left[ \frac{\cos \left( \hat{n},\hat{r}\right) -\cos \left( \hat{n}, \hat{\rho }\right) }{2}\right] dS. \end{aligned}$$

In Eq. (6), *S* is the aperture the wave passes through and $f_S$ is the complex aperture function over the surface *S*. The unit vector $\hat{n}$ is the normal vector on the infinitesimal surface element *dS*. The scalars $\rho = \left| \vec {\rho } \right|$ and $r = \left| \vec {r} \right|$ denote the distance from the source *s* to the surface element *dS* and from *dS* to *P*, respectively, as shown in Fig. 4. The vectors $\hat{r}, \hat{\rho }$ are the unit vectors of $\vec {r}$ and $\vec {\rho }$, respectively. Although the diffraction formula deals with transmission through the surface *S*, it can also be applied to calculate the electric field of the reflected waves from *S*, as is true for the RIS. In this case, the surface *S* is the aperture of the RIS and the complex aperture function $f_S$ is equivalent to the complex $S_{31}$-parameters of the RIS unit cells, i.e. $f_S=S_{31}$.

In good approximation, we assume a constant aperture function $f_S$ over each individual RIS unit cell, which allows us to express the integral over the whole RIS as a sum of integrals resulting in the electric field $\mathscr {E}_P$ at point P

7 $$\begin{aligned} \mathscr {E}_P =\sum _{(m,n)} \left[ f_{(m,n)} \cdot \underbrace{\int _{S_{(m,n)}}\frac{\exp \left\{ ik(\rho +r)\right\} }{\rho r}\cdot \left[ \frac{\cos \left( \hat{n},\hat{r}\right) -\cos \left( \hat{n}, \hat{\rho }\right) }{2}\right] dS_{(m,n)}}_{k_{p(m,n)}}\right] , \end{aligned}$$

where (*m*, *n*) indexes the unit cells of the RIS. According to Eq. (5), the complex aperture function of each unit cell equals $f_{(m,n)} = a_{(m,n)}\exp \left\{ j\varphi _{(m,n)}\right\}$, with the attenuation $a_{(m,n)}$ and phase shift $\varphi _{(m,n)}$ of the corresponding RIS unit cell (*m*, *n*).

In Eq. (7), the integral term $k_{p{(m,n)}}$ does not depend on the configuration $f_S$ of the $(m,n)^\text {th}$ unit cell, and can be precomputed. In matrix form, this yields

8 $$\begin{aligned} \textbf{K}_P = \left[ \begin{array}{ccc} k_{p(1,1)} & \dots & k_{p(1,N)}\\ \vdots & \ddots & \vdots \\ k_{p(M,1)} & \dots & k_{p(M,N)}\\ \end{array}\right] . \end{aligned}$$

With the complex aperture function matrix of the RIS

9 $$\begin{aligned} \textbf{F} = \left[ \begin{array}{ccc} f_{(1,1)} & \dots & f_{(1,N)}\\ \vdots & \ddots & \vdots \\ f_{(M,1)} & \dots & f_{(M,N)}\\ \end{array}\right] , \end{aligned}$$

we can express Eq. (7) as a sum over the component-wise matrix product of $\textbf{K}_P$ and $\textbf{F}$, which can be computed efficiently for each field observation point *P* as

10 $$\begin{aligned} \mathscr {E}_P = \sum _{(m,n)} \textbf{F} \circ \textbf{K}_P. \end{aligned}$$

It can be readily seen that the maximum electric field strength in a point *P* is reached for

11 $$\begin{aligned} \textbf{F} = \textbf{K}_P^*, \end{aligned}$$

which guarantees constructive interference due to $\arg \left( \textbf{K}_P^*\right) = -\arg \left( \textbf{K}_P\right)$ and consequently $\arg \left( \textbf{K}_P^*\circ \textbf{K}_P\right) = 0$.

### Machine learning model and training process

According to Eq. (11), beam steering to a defined position *P*, and thus maximization of $\mathscr {E}_P$, requires that $\textbf{F} = \textbf{K}_P^*$. In our case however, in which we want to steer two beams with two different carrier frequencies to two different positions $P_I$ and $P_{II}$ at the same time, we have to follow this optimization process to maximize the fields $\mathscr {E}_{P_I}$ and $\mathscr {E}_{P_{II}}$ at the carrier frequencies $\nu _1 = {27}\hbox {Hz}$ and $\nu _2 = {31}\hbox {GHz}$ simultaneously. As an additional constraint, the attenuation and the phase of the RIS can only be tuned according to the bias voltage characteristics in Fig. 3b, where phase shift and attenuation are not independent of each other and limited in range. To find proper solutions for the non-trivial optimization problem, we implemented a machine learning architecture to directly optimize for the bias voltage patterns that must be applied to the varactor diodes on the RIS for simultaneous two-colour beam steering. This optimization process will be described in the following.

**Fig. 5:**
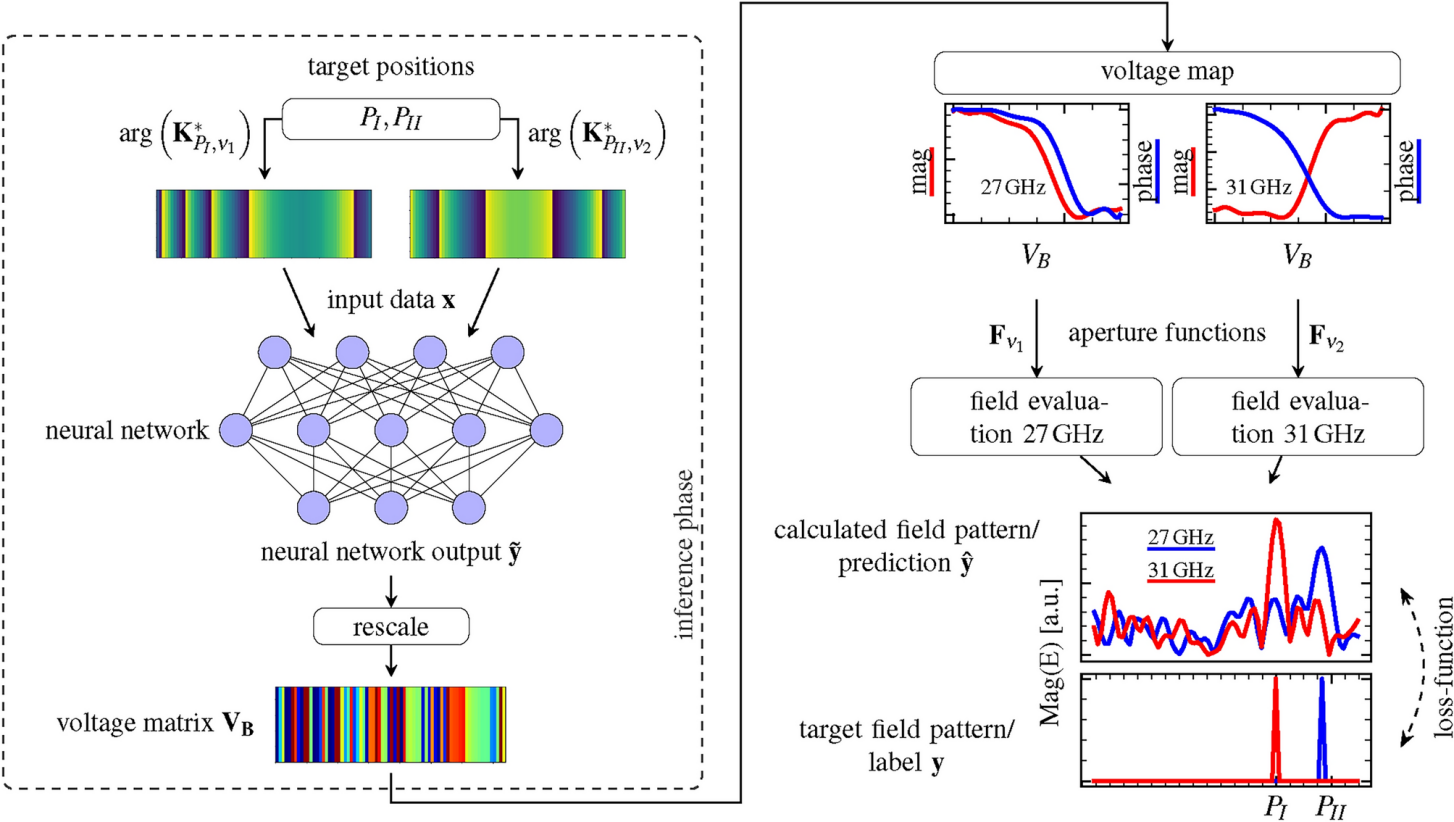
Machine learning architecture. The phases $\arg \left( \textbf{K}_{P_I,\nu _1}^*\right)$ and $\arg \left( \textbf{K}_{P_{II},\nu _2}^*\right)$ at the target deflection points $P_I$ and $P_{II}$ are used as input to a neural network that computes the bias voltage vector for the RIS. From the bias voltage matrix, spatial electric field distribution in the observation plane is calculated via the voltage map- and the field evaluation-layer. The loss function of the machine learning architecture compares this electric field distribution $\hat{\textbf{y}}$ to the target field pattern $\textbf{y}$ by categorical cross-entropy to provide a gradient for the training process of the neural network.

Figure 5 shows the schematic of the optimization algorithm. The core of the machine learning architecture is a fully-connected neural network with three hidden layers of 148 neurons each.

The input vector $\textbf{x}$ of the network consists of the concatenation of the vectors $\arg \left( \textbf{K}_{P_I, \nu _1}^*\right)$ and $\arg \left( \textbf{K}_{P_{II}, \nu _2}^*\right)$, calculated for the points $P_I$ and $P_{II}$ at the frequencies $\nu _1$ and $\nu _2$ according to Eq. (11):

12 $$\begin{aligned} \textbf{x} = \left( \arg \left( \textbf{K}_{P_I, \nu _1}^*\right) ,\arg \left( \textbf{K}_{P_{II}, \nu _2}^*\right) \right) \end{aligned}$$

The reason for feeding the neural network with phase information only is that the phase carries all substantial information, while the corresponding magnitudes are all close to one.

The dimensions of $\arg \left( \textbf{K}_{P_I, \nu _1}^*\right)$ and $\arg \left( \textbf{K}_{P_{II}, \nu _2}^*\right)$ are $(74\times 1)$ for both, as we account for the unstructured perimeter (two unit cells in width) of each module and the one unit cell-wide gaps between the modules (see Fig. 3a .) This yields a dimension of the input vector of $\dim (\textbf{x}) = (148\times 1)$.

For activation, we used a hyperbolic-tangent function to ensure that the output vector $\tilde{\textbf{y}}$ of the neural network assumes values between $-1$ and 1 that we rescaled by $\mathbf {V_B} = (\tilde{\textbf{y}} \cdot {15}\hbox {V}) - {5}\hbox {V}$ to obtain the correct bias voltage vector. We then inserted $\mathbf {V_B}$ into Eq. (5) to compute the complex aperture functions for the frequencies $\nu _1$ and $\nu _2$ from the voltage map according to

13 $$\begin{aligned} \begin{aligned} \textbf{F}_{\nu _1}&= \tilde{a}_{\nu _1}(\mathbf {V_B}) \exp \left\{ j\frac{\pi }{180}\tilde{\varphi }_{\nu _1}(\mathbf {V_B})\right\} \\ \textbf{F}_{\nu _2}&= \tilde{a}_{\nu _2}(\mathbf {V_B}) \exp \left\{ j\frac{\pi }{180}\tilde{\varphi }_{\nu _2}(\mathbf {V_B})\right\} \end{aligned} \end{aligned}$$

In the subsequent field evaluation layer, we applied Eq. (10) to calculate the electric field at the points $P_i$ in the observation plane, with $i=1\ldots L=91$, resulting in an angular resolution of $0.5^\circ$ when computing the fields in an angular range $\phi \in [0^\circ , 45^\circ ]$ by

14 $$\begin{aligned} \begin{aligned} \mathscr {E}_{P_i,\nu _1} = \sum _{(m,n)} \textbf{F}_{\nu _1} \circ \textbf{K}_{P_i,\nu _1}\\ \mathscr {E}_{P_i,\nu _2} = \sum _{(m,n)} \textbf{F}_{\nu _2} \circ \textbf{K}_{P_i,\nu _2}\\ \text {with} \quad i=1,\dots L. \end{aligned} \end{aligned}$$

Equation (14) yields the prediction vector $\hat{\textbf{y}}$ of the machine learning architecture, i.e. the spatial electric field distribution in the observation plane for the frequencies $\nu _1$ and $\nu _2$:

15 $$\begin{aligned} \begin{aligned} \hat{\textbf{y}}&= \left( \left[ \mathscr {E}_{P_1,\nu _1}, \dots \mathscr {E}_{P_L,\nu _1}\right] ^T, \right. \\&\quad \left. \left[ \mathscr {E}_{P_1,\nu _2} , \dots \mathscr {E}_{P_L,\nu _2}\right] ^T\right) \end{aligned} \end{aligned}$$

The neural network is trained by generating random samples of deflection angle pairs $(\phi _1,\phi _2)$, that correspond to points $(P_I,P_{II})$ on the screen at a distance *r*, and inserting the according phases $\left( \arg \left( \textbf{K}_{P_I,\nu _1}^*\right) ,\arg \left( \textbf{K}_{P_{II},\nu _2}^*\right) \right)$ as input vector $\textbf{x}$. The target electric field patterns, i.e. the labels $\textbf{y}$ of the training data, are sparse vectors that only contain non-zero entries at the target points $P_I$ and $P_{II}$ of the deflection, where the values are 1. We obtain

16 $$\begin{aligned} \begin{aligned} \textbf{y}&= \left( \left[ 0,\dots ,y_{(P=P_I)}=1,0,\dots \right] ^T, \right. \\&\quad \left. \left[ 0,\dots ,y_{(P = P_{II})}=1,0,\dots \right] ^T\right) \end{aligned} \end{aligned}$$

The prediction vector $\hat{\textbf{y}}$ is then compared to the target vector $\textbf{y}$ by calculating the categorical cross-entropy loss function. This provides a gradient for training the neural network. After convergence, the neural network can be used to predict the required bias voltage vector that must be applied to the RIS for dynamically tunable, simultaneous, frequency-selective beam steering of an incident beam to two different moving positions. In our two-color beam steering application, the training converged after 200 epochs with a training dataset size of 32.000 samples of uniformly distributed target deflection angle pairs between $0^\circ$ and $45^\circ$, resulting in 6.4 million field evaluations overall.

## Beam steering results and verification

In the first part of this section, we describe the measurement setup used to evaluate the proposed method. Subsequently, we discuss the experimental verification of two-color beam steering with bias voltage patterns obtained from our machine learning process. Last, we compare the channel capacity in MU-FSBS to the corresponding capacity in TDMA, as described in section "Multi user-frequency selective beam steering (MU-FSBS)".

### Measurement setup

**Fig. 6:**
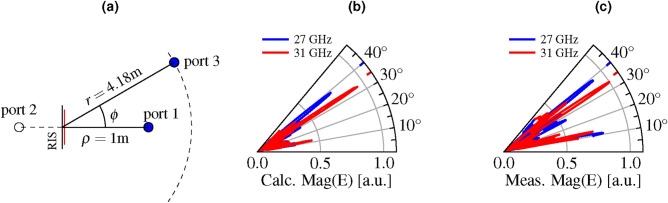
Beam steering measurements. (**a**) Schematic setup for measuring beam steering in one plane. The RIS is placed in the center of a one-dimensional goniometer of radius 4.18 m. The transmitter antenna (port 1) is placed at a distance of ${1}\,\hbox {m}$ orthogonally to the RIS. The receiver antenna (port 3) can move along the goniometer axis from $10^\circ$ to $45^\circ$. (**b**) Calculated and (**c**) measured electric field distribution for a target deflection angle pair $(\phi _1,\phi _2) = (35^\circ , 40^\circ$), normalized to the maximum of the field that was measured within the target angle range from 10$^\circ$ to 45$^\circ$.

To measure the electric field created by the RIS for specific bias voltage patterns, we placed the RIS in the center of a one-dimensional goniometer with a radius of $4.18\hbox {m}$ as described schematically in Fig. 6a. We describe the RIS as a 3-port device where port 1 and port 3 are defined at the coaxial connector of the transmitting and receiving horn antenna, respectively. Port 2 is defined behind the RIS, however we do not actually measure at this port because the transmission through the RIS is negligible and not of interest. The transmitting horn antenna is placed orthogonally to the RIS at a distance of 1 m, while the receiving horn antenna can move along the goniometer axis from $+10^\circ$ to $+45^\circ$. The $S_{31}$ scattering parameter from the transmitting antenna (port 1) to the receiving antenna (port 3) is measured with a vector network analyzer (Rohde & Schwarz ZNA43). The influence of parasitic reflections from the environment is minimized by microwave absorbers behind the RIS and by time-gating the $S_{31}$-signal, to receive only reflections within a window of 7 ns around the expected arrival time of the RIS’s reflection.

### Electric field distributions with MU-FSBS

We demonstrate synchronous, frequency-dependent beam steering of a normally incident beam to target deflection angles $\phi _1, \phi _2 \in \left[ 10^\circ , 45^\circ \right]$ at the corresponding carrier frequencies $\nu _1={31}\, \hbox {GHz}$ and $\nu _2={27}\, \hbox {GHz}$ by applying the optimized bias voltage patterns to the RIS and measuring the electric field distributions in the goniometer. In total, we sampled all the possible combinations of target deflection angles $(\phi _1,\phi _2) \in [10^\circ ,45^\circ ]$ on a $5^\circ$ grid. Figure 6 shows an example of the calculated and measured electric field strengths for the target deflection angle pair $\phi _1=35^\circ$ at ${31}\, \hbox {GHz}$ and $\phi _2=40^\circ$ at ${27}\, \hbox {GHz}$. The electric field amplitude was normalized to the maximum electric field amplitude that was measured within the target deflection angle range from 10$^\circ$ to 45$^\circ$. As expected, the electric field peaks at the desired target deflection angle for the corresponding carrier frequency.

Compared to the electric field that is backscattered from an unstructured aluminum plate of the same size and in the same position as the RIS, i.e. normal to the incident wave, the signal power at the target position increased by approximately 13.5 dB at a deflection angle of $35^\circ$ and by about 18.5 dB at a deflection angle of $40^\circ$. The statistic average over all the measured combinations of deflection angles reveals an increase in signal power by at least ${5.5}\,\hbox {dB}$ and up to ${28.4}\,\hbox {dB}$ with an average increase of ${17.15}\,\hbox {dB}$ at the target deflection angles.

The power peak at $\phi = 10^\circ$ originates from diffraction at the aperture of the RIS (see Fig. 3a ) and occurs in all measurements, regardless of the applied bias voltage pattern and even in the measurements of the reference aluminum plates.

In the supplementary material to this publication, we present a numerical investigation of MU-FSBS in a three user-scenario.

### MU-FSBS vs. TDMA: channel capacity and critical SNR

For investigating the dependence of the channel capacity on the SNR in MU-FSBS and TDMA in the present example configuration, we used the machine learning architecture to determine the required bias voltage patterns for target deflection pairs $(\phi _1,\phi _2) \in [10^\circ ,45^\circ ]$ on a $5^\circ$ grid in MU-FSBS. Remember that the deflection angle pair corresponds to the carrier frequency pair $({31}\, \hbox {GHz}$,${27}\, \hbox {GHz})$. In TDMA, we optimized the required bias voltage vectors by means of an efficient particle swarm algorithm, which is known as a very reliable global optimization method. For the determination of the critical SNR$_{crit}$, according to the definition in section "Multi user-frequency selective beam steering (MU-FSBS)", we measured the beam power in the main lobes for all deflection angle combinations in both MU-FSBS and TDMA and derived the critical signal to noise ratio $\text {SNR}_\text {crit}$. Remember that the critical SNR$_{crit}$ in Eq. (4) was defined as the minimum required signal power normalized to the noise power, for which the channel capacity $C_\text {MU}$ in MU-FSBS is equal or higher than the channel capacity $C_\text {TD}$ in TDMA.

**Fig. 7:**
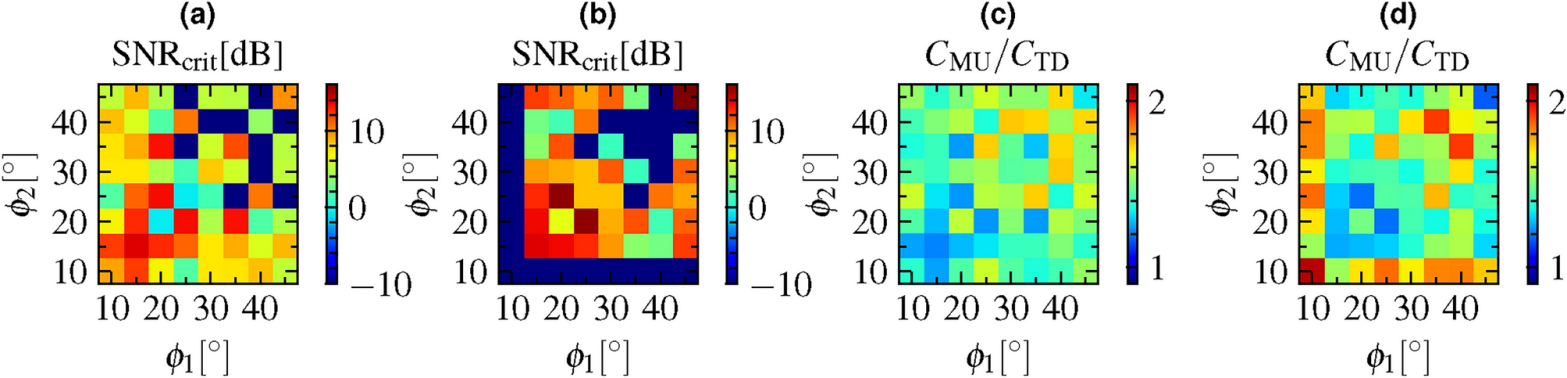
Experimental comparison of MU-FSBS and TDMA. Critical signal-to-noise ratio ($\text {SNR}_\text {crit}$), for which the total channel capacity in MU-FSBS is equal or higher than in (**a**) TDMA at a carrier frequency of ${27}\, \hbox {GHz}$ and (**b**) TDMA at ${31}\, \hbox {GHz}$. $\text {SNR}_\text {crit}$ values of less than -10 dB have been clipped to -10 dB for the sake of contrast. The ratio $C_{MU}/C_{TD}$ of the channel capacities in MU-FSBS and TDMA at a TDMA carrier frequency of (**c**) ${27}\, \hbox {GHz}$ and (**d**) ${31}\, \hbox {GHz}$ for a fixed SNR $S_0/N = {20}\hbox {dB}$.

Figure 7a and 7b show color maps of the critical SNR$_{crit}$ for the operation frequencies of ${27}\, \hbox {GHz}$ and ${31}\, \hbox {GHz}$ dependent on the target deflection angle pair $(\phi _1,\phi _2)$. Hereby, $\phi _1$ and $\phi _2$ are denoted on the abscissa and the ordinate, respectively. For MU-FSBS, the signal powers at the target deflection angle pairs were not equal, but optimized to obtain the maximum sum signal power. For some combinations of target deflection angles the signal powers in the two deflection arms in MU-FSBS exceed the corresponding signal powers in TDMA, which means that $\text {SNR}_\text {crit}$ diverges to $-\infty$ dB. For the sake of better contrast in the color plots, these values have been clipped to ${-10}\,\hbox {dB}$.

Over all combinations of target deflection angle pairs $(\phi _1,\phi _2)$, the critical SNR$_{crit}$ compared to TDMA at 27 GHz reaches a maximum value of 13.84 dB and a median of 5.95 dB. Compared to TDMA at 31 GHz the maximum critical SNR$_{crit}$ is at 16.01 dB and its median is at 2.88 dB. Such SNR are generally common in communication scenarios. In our laboratory setup for example, we measured SNRs of ${61.71}\,\hbox {dB}$ for the ${27}\, \hbox {GHz}$ band and ${61.54}\,\hbox {dB}$ for the ${31}\, \hbox {GHz}$ band for the exemplary field pattern shown in Fig. 6.

Simulation studies on ${28}\, \hbox {GHz}$ mm-Wave networks in^30^ suggest that path loss values below $110\,\text {dB}$ can be obtained in LOS areas, corresponding to SNRs up to ${30}\,\hbox {dB}$ when accounting for all gains and losses given in the link budget for this scenario. Additional simulations in mm-Wave networks and measurement studies in sub-6 GHz 5G campus networks support that these are indeed attainable SNR values in real world LOS-communication scenarios^31–33^.

A more application-oriented perspective on the benefit of MU-FSBS over TDMA is to evaluate the ratio of channel capacities $C_\text {MU}/C_\text {TD}$ for a fixed SNR. Figure 7c and 7d show the dependence of the ratio $C_\text {MU}/C_\text {TD,27\,GHz}$ at a TDMA carrier frequency of 27 GHz and $C_\text {MU}/C_\text {TD,31\,GHz}$ at 31 GHz on the deflection angle pair $(\phi _1,\phi _2)$. In this case, the SNR was ${20}\,\hbox {dB}$. Compared to TDMA at ${27}\, \hbox {GHz}$, MU-FSBS offers an average increase of the sum channel capacity by a factor of 1.474 and a minimum increase by a factor of 1.254. Compared to TDMA at ${31}\, \hbox {GHz}$, we observe an increase of channel capacity by 1.533 on average and at minimum by 1.161. Averaged over both frequencies, MU-FSBS promises approximately ${50}\,{\%}$ higher channel capacity than TDMA for a SNR of ${20}\,\hbox {dB}$.

Table 1 lists the average and minimum channel capacity increase in MU-FSBS versus TDMA for SNR values from ${10}\,\hbox {dB}$ to ${60}\,\hbox {dB}$ that cover the typical SNRs in the aforementioned studies. Even for a SNR of ${10}\,\hbox {dB}$, we expect an increase in channel capacity by ${13.9}\,{\%}$ on average, although there are some combinations of deflection angles for which TDMA can achieve higher channel capacity than MU-FSBS when the SNR is below ${15}\,\hbox {dB}$. However, above a SNR of ${20}\,\hbox {dB}$, MU-FSBS promises higher channel capacity for any combination of deflection angles on the evaluated grid.

**Table 1:** Evaluation of increase in channel capacity ($C_{MU}/C_{TD} - 1$ [$\,{\%}$]) of MU-FSBS compared to TDMA at ${27}\, \hbox {GHz}$ and ${31}\, \hbox {GHz}$ for different SNRs.

SNR [$\hbox {dB}$]	MU-BS vs. ${27}\, \hbox {GHz}$ TDMA		MU-BS vs. ${31}\, \hbox {GHz}$ TDMA	
	average [%]	min [%]	average [%]	min [%]
10	13.9	-17.7	24.0	-25.2
15	32.3	5.3	39.9	-4.3
20	47.4	25.4	53.3	16.1
25	58.3	40.3	63.0	32.4
30	65.8	50.8	69.8	44.5
35	71.2	58.3	74.5	53.2
60	84.0	76.4	85.9	74.1

## Conclusion

We presented multi-user frequency selective beam steering (MU-FSBS) that potentially increases the available channel capacity, when a multi-carrier signal is sent over a RIS to serve multiple user areas at different, time-varying locations simultaneously. The development of a custom neural network-based machine learning architecture enabled us to generate non-trivial bias-voltage patterns for tuning the frequency-selective deflection of an incident beam from a varactor-based RIS. In our exemplary experimental demonstration in the Ka-band, we simultaneously converted an incident wave with carrier frequencies $\nu _1$ and $\nu _2$ into two separate beams, one with carrier frequency $\nu _1$ and the other with carrier frequency $\nu _2$ and dynamically steered them independently to two different, varying locations at the same time. In our example, the carrier frequencies were $\nu _1=$27 GHz and $\nu _2=$31 GHz and the achieved deflection angles for both beams were in the range from $10^\circ$ and $45^\circ$ each. We also compared MU-FSBS to TDMA and found an average channel capacity increase in MU-FSBS by approximately $50\%$ for a fixed SNR of ${20}\,\hbox {dB}$. Hereby, we assumed that the channel bandwidth in all used frequency bands were equal. For a SNR of ${60}\,\hbox {dB}$, MU-FSBS promises $84\%$ higher channel capacity than TDMA under the aforementioned assumption.

## Supplementary Information

Supplementary Information.

## Data Availability

The data that support the findings of this study are available from the corresponding author upon reasonable request.
