# Supplementary material for: Multi-user frequency selective beam steering by reconfigurable intelligent surfaces in the Ka-band
Source: Sci Rep. 2025 Mar 29;15:10891. doi: 10.1038/s41598-025-95063-1 (PMC11954988; doi:10.1038/s41598-025-95063-1)
Supplement: Supplementary file 1 — Supplementary Information. [file 41598_2025_95063_MOESM1_ESM.pdf]

Supplementary Material to:
Multi-User Frequency Selective Beam Steering by Reconfigurable Intelligent Surfaces in the Ka-Band

Lukas Mueller[1,+], Alexander Wolff[1,+,*], Steffen Klingel[1], Janis Krieger[1], Lars Franke[1], Ralf Stemler[1], and Marco Rahm[1,*]

[1]RPTU Kaiserslautern-Landau, Department of Electrical and Computer Engineering and Research Center OPTIMAS, Kaiserslautern, D-67663, Germany
[*]marco.rahm@rptu.de, alexander.wolff@rptu.de
[+]these authors contributed equally to this work

Machine Learning Model for an Arbitrary Number of Users

Multi-User Frequency Selective Beam Steering (MU-FSBS) can be readily generalized to more than two users. In this supplementary document, we describe the machine learning model from section 4.2 in a generalized form that allows for N users.

According to equation (11), beam steering to a defined position P, and thus maximization of $\mathscr{E}_P$, requires that $\mathbf{F} = \mathbf{K}_P^*$. In our case however, in which we want to steer N beams with N different carrier frequencies to N different positions $(P_I, P_{II}, ..., P_N)$, at the same time, we have to follow this optimization process to maximize the fields $\mathscr{E}_{P_I}$ to $\mathscr{E}_{P_N}$ at the carrier frequencies $(\nu_1, \nu_2, ..., \nu_N)$ simultaneously. As an additional constraint, the attenuation and the phase of the RIS can only be tuned according to the bias voltage characteristics in fig. 3b and fig. S2a, where phase shift and attenuation are not independent of each other and limited in range. To find proper solutions for the non-trivial optimization problem, we implemented a machine learning architecture to directly optimize for the bias voltage patterns that must be applied to the varactor diodes on the RIS for simultaneous N-colour beam steering. This optimization process will be described in the following.

Figure S1 shows the schematic of the generalized optimization algorithm. The core of the machine learning architecture is a fully-connected neural network with three hidden layers. The input layer and each hidden layer of the neural network comprise $N \times M$ neurons, where M is the number of individually tunable unit cell columns of the RIS. For a higher number of beams N, eventually, the number of hidden layers may have to be increased. For $N = 3$ beams, however, 3 hidden layers are sufficient, as will be demonstrated in the example of the following section.

The input vector $\mathbf{x}$ of the neural network consists of the concatenation of the vectors $\arg\left(\mathbf{K}^*_{P_I,\nu_1}\right)$ to $\arg\left(\mathbf{K}^*_{P_N,\nu_N}\right)$ calculated for the points P_I to P_N at the frequencies ν_1 to ν_N according to equation (11):

$$\mathbf{x} = \left(\arg\left(\mathbf{K}^*_{P_I,\nu_1}\right)^T, \arg\left(\mathbf{K}^*_{P_{II},\nu_2}\right)^T, \ldots, \arg\left(\mathbf{K}^*_{P_N,\nu_N}\right)^T\right) \tag{S1}$$

The dimensions of $\arg\left(\mathbf{K}^*_{P_I,\nu_1}\right)$ to $\arg\left(\mathbf{K}^*_{P_N,\nu_N}\right)$ are $(M \times 1)$, which yields a dimension of the input vector of $\dim(\mathbf{x}) = (N \times M \times 1)$.

For activation, we used a hyperbolic-tangent function to ensure that the output vector $\tilde{\mathbf{y}}$ of the neural network assumes values between -1 and 1 that we rescaled by $\mathbf{V_B} = (\tilde{\mathbf{y}} \cdot 15\,\mathrm{V}) - 5\,\mathrm{V}$ to obtain the correct bias voltage vector. We then inserted $\mathbf{V_B}$ into equation (5) to compute the complex aperture functions for the frequencies ν_1 to ν_N from the voltage map according to

$$\begin{aligned}
\mathbf{F}_{\nu_1} &= \tilde{a}_{\nu_1}(\mathbf{V_B}) \exp\left\{j\frac{\pi}{180}\tilde{\varphi}_{\nu_1}(\mathbf{V_B})\right\} \\
\mathbf{F}_{\nu_2} &= \tilde{a}_{\nu_2}(\mathbf{V_B}) \exp\left\{j\frac{\pi}{180}\tilde{\varphi}_{\nu_2}(\mathbf{V_B})\right\} \\
&\vdots \\
\mathbf{F}_{\nu_N} &= \tilde{a}_{\nu_N}(\mathbf{V_B}) \exp\left\{j\frac{\pi}{180}\tilde{\varphi}_{\nu_N}(\mathbf{V_B})\right\}
\end{aligned} \tag{S2}$$

In the subsequent field evaluation layer, we applied equation (10) to calculate the electric field at the points P_i in the observation plane, with $i = 1 \ldots L$ as follows:

$$\begin{aligned}
\mathscr{E}_{P_i,\nu_1} &= \sum_{(m,n)} \mathbf{F}_{\nu_1} \circ \mathbf{K}_{P_i,\nu_1} \\
\mathscr{E}_{P_i,\nu_2} &= \sum_{(m,n)} \mathbf{F}_{\nu_2} \circ \mathbf{K}_{P_i,\nu_2} \\
&\vdots \\
\mathscr{E}_{P_i,\nu_N} &= \sum_{(m,n)} \mathbf{F}_{\nu_N} \circ \mathbf{K}_{P_i,\nu_N} \\
&\text{with} \quad i = 1, \ldots L.
\end{aligned} \tag{S3}$$

Here, L is the number of electric field samples. The prediction vector $\hat{\mathbf{y}}$ of the machine learning architecture, i.e. the desired spatial electric field distribution in the observation plane for the frequencies ν_1 to ν_N, is described by:

$$\hat{\mathbf{y}} = \left([\mathscr{E}_{P_1,\nu_1}, \ldots \mathscr{E}_{P_L,\nu_1}]^T, [\mathscr{E}_{P_1,\nu_2}, \ldots \mathscr{E}_{P_L,\nu_2}]^T, \ldots, [\mathscr{E}_{P_1,\nu_N}, \ldots \mathscr{E}_{P_L,\nu_N}]^T \right) \tag{S4}$$

The neural network is trained by generating random samples of deflection angle tuples $(\phi_1, \phi_2, \ldots, \phi_N)$, that correspond to points $(P_I, P_{II}, \ldots, P_N)$ in the field evaluation plane, and inserting the according phases as input vector $\mathbf{x}$ as described in equation (S1). The target electric field patterns, i.e. the labels $\mathbf{y}$ of the training data, are sparse vectors that only contain non-zero entries at the target points P_I to P_N of the deflection, where the values are 1, which yields

$$\mathbf{y} = \left([0, \ldots, y_{(P=P_I)} = 1, 0, \ldots]^T, [0, \ldots, y_{(P=P_{II})} = 1, 0, \ldots]^T, \ldots, [0, \ldots, y_{(P=P_N)} = 1, 0, \ldots]^T \right) \tag{S5}$$

The prediction vector $\hat{\mathbf{y}}$ is then compared to the target vector $\mathbf{y}$ by calculating the categorical cross-entropy loss function. This provides a gradient for training the neural network. After convergence, the neural network can be used to predict the required bias voltage vector that must be applied to the RIS for dynamically tunable, simultaneous, frequency-selective beam steering of an incident beam to N different moving positions.

Numerical Evaluation for the Example of Three-User Frequency Selective Beam Steering

As an example for the scalability of MU-FSBS to more than two users, we numerically validated three-user frequency selective beam steering. Here, we used a RIS of 40×40 unit cells, which was capable to steer the beam in the two dimensions of a plane. The reflection coefficient of the unit cell of this RIS was identical with the one presented in section 3. As operating frequencies, we chose 27 GHz, 28 GHz and 31 GHz for the three users. Figure S2a illustrates the obtained attenuation and phase shift of the unit cell at the three operating frequencies towgether with 10th order polynomial fitting curves. For the following analysis, the center of the RIS was defined as the origin of a cartesian coordinate system, as depicted in fig. S2b. The electric field was evaluated in the plane parallel to the XZ-plane at $y = -250$ mm. After the training of the neural network for three users, we chose three exemplary combinations of target positions (P_I, P_{II}, P_{III}) randomly within the field evaluation plane and calculated the optimal bias voltage patterns and the expected electric field distributions for these tuples. In rows (a) and (b) of fig. S3, fig. S4 and fig. S5, we illustrated the magnitude $\tilde{a}$ and phase $\tilde{\varphi}$ of the reflection coefficient that corresponds to the optimized bias voltage patterns $\mathbf{V_B}$. Furthermore, we illustrated the calculated electric field distributions in row (c) of of the same figures. We observe that in each of the three example cases, MU-FSBS yields electric field maxima at the target positions, as expected.

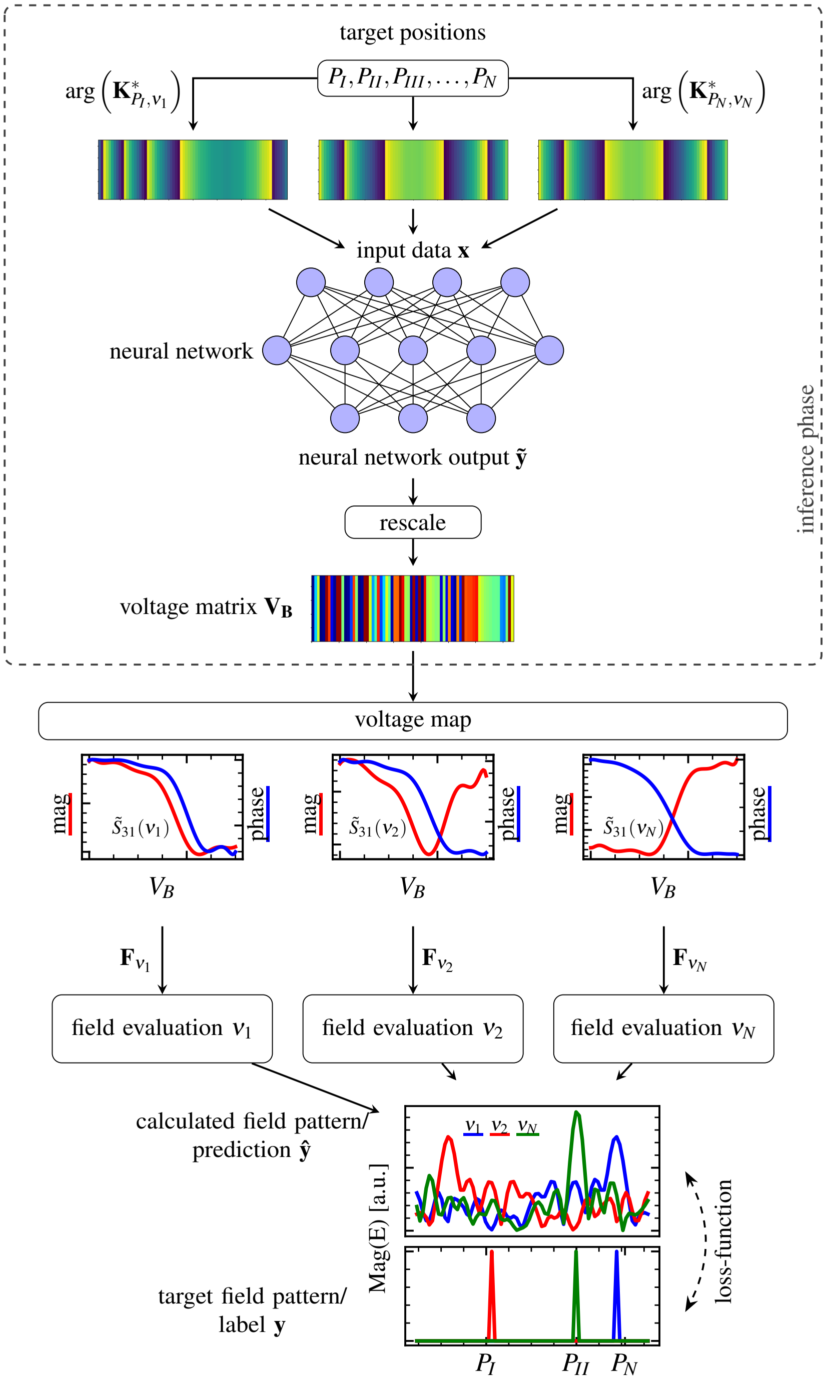

Figure S1. Machine learning architecture. The phases $\arg\left(\mathbf{K}^*_{P_I,\nu_1}\right)$ to $\arg\left(\mathbf{K}^*_{P_N,\nu_N}\right)$ at the target deflection points P_I to P_N are used as input to a neural network that computes the bias voltage vector for the RIS. From the bias voltage matrix, the spatial electric field distribution in the observation plane is calculated via the voltage map and the field evaluation layer. The loss function of the machine learning architecture compares this electric field distribution $\hat{\mathbf{y}}$ to the target field pattern $\mathbf{y}$ by categorical cross-entropy to provide a gradient for the training process of the neural network.

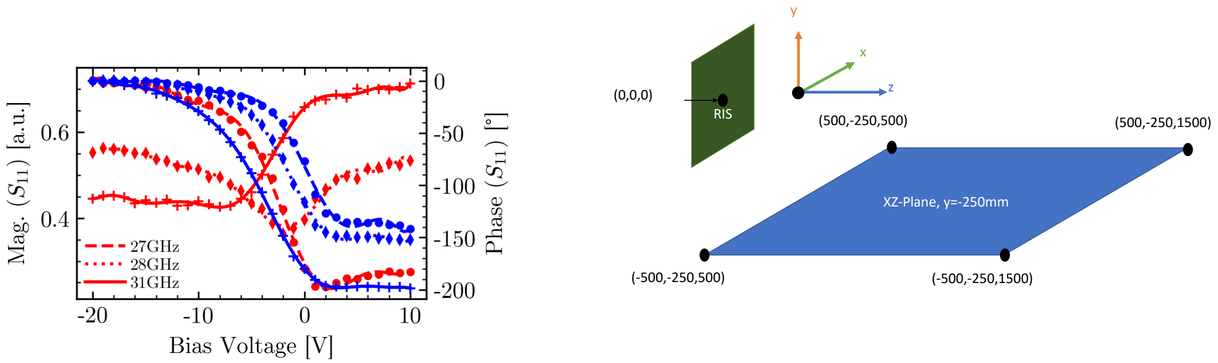

(a) **(b)**

Figure S2. a RIS characterization. Attenuation a and phase shift φ of the RIS unit cell for 27 GHz (circles), 28 GHz (diamonds) and 31 GHz (crosses) at the measured bias voltages. The lines indicate 10th order polynomial fits for the attenuation $\tilde{a}$ and the phase shift $\tilde{\varphi}$ of the RIS unit cell. b Scenario for MU-FSBS with three users. The RIS constists of 40×40 unit cells. We evaluate the electric field in the plane at $y = -250$ mm.

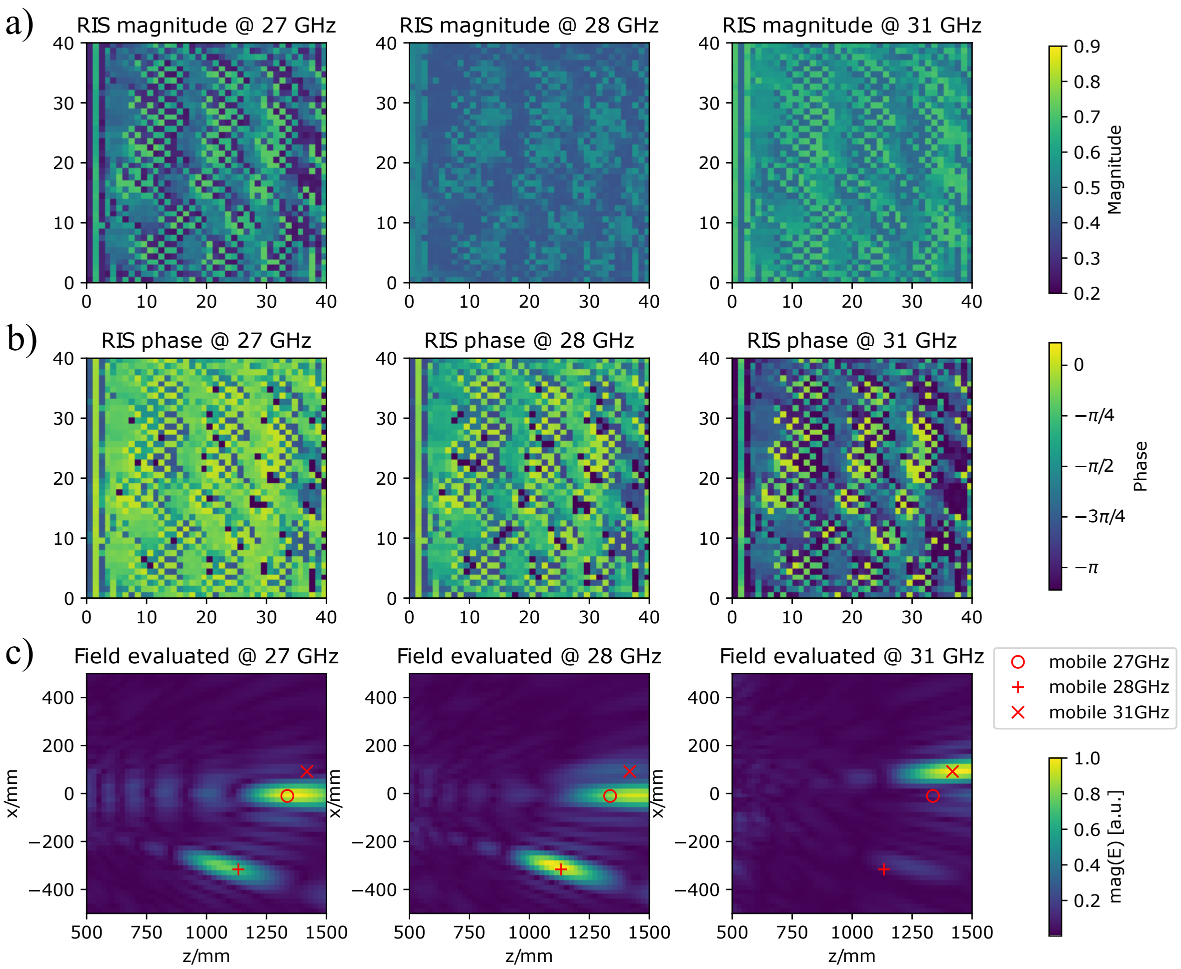

Figure S3. 1st exemplary configuration of a three-user scenario: The three columns correspond to the three working frequencies at 27 GHz, 28 GHz and 31 GHz. (a) magnitudes of the reflection coefficient for the optimized bias voltage, (b) Respective phases of the reflection coefficients, (c) Calculated electric fields in arbitrary units and the target positions of the foci for the three user frequencies. We observe that MU-FSBS can create foci at each of the target positions as expected.

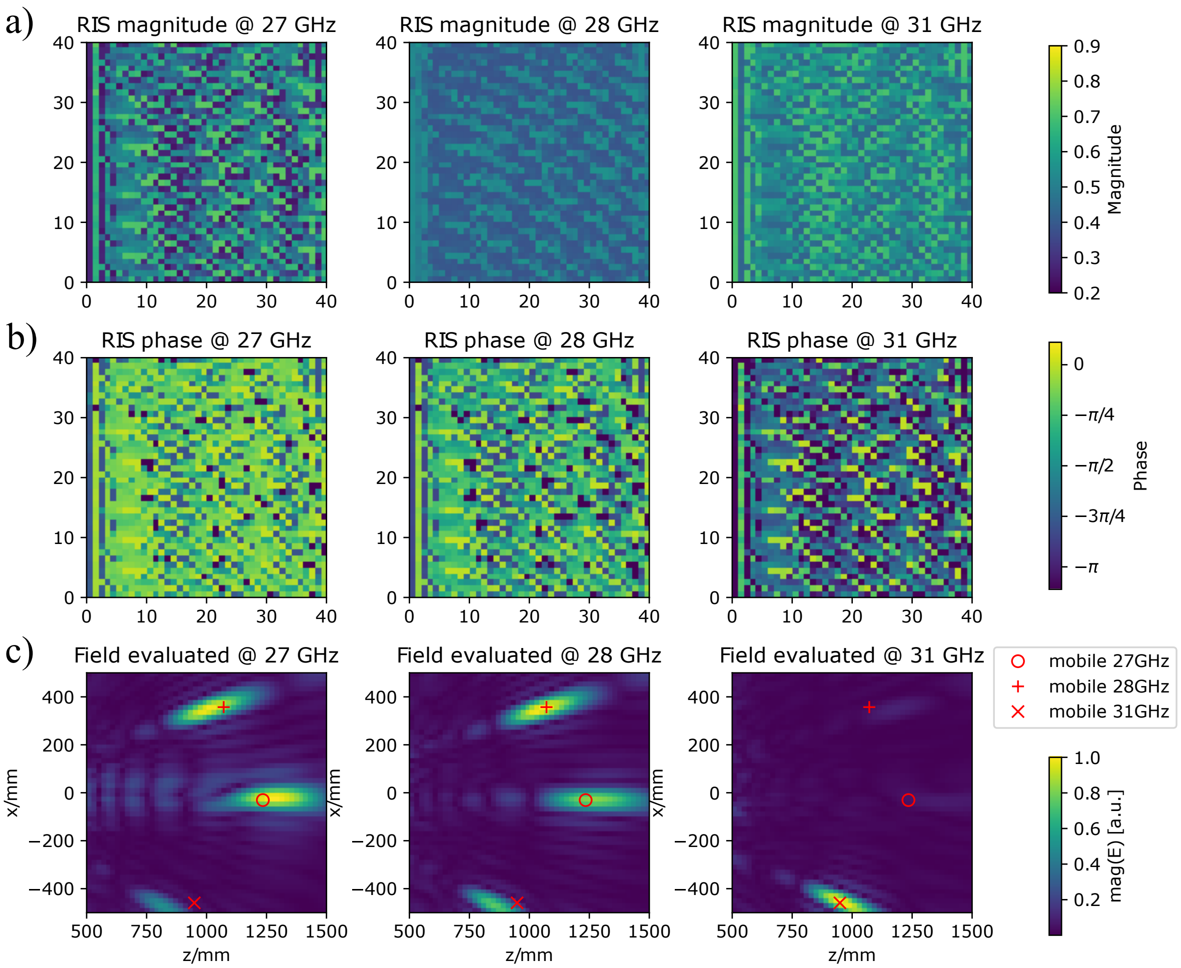

Figure S4. 2nd exemplary configuration of a three-user scenario: The three columns correspond to the three working frequencies at 27 GHz, 28 GHz and 31 GHz. (a) magnitudes of the reflection coefficient for the optimized bias voltage, (b) Respective phases of the reflection coefficients, (c) Calculated electric fields in arbitrary units and the target positions of the foci for the three user frequencies. We observe that MU-FSBS can create foci at each of the target positions as expected.

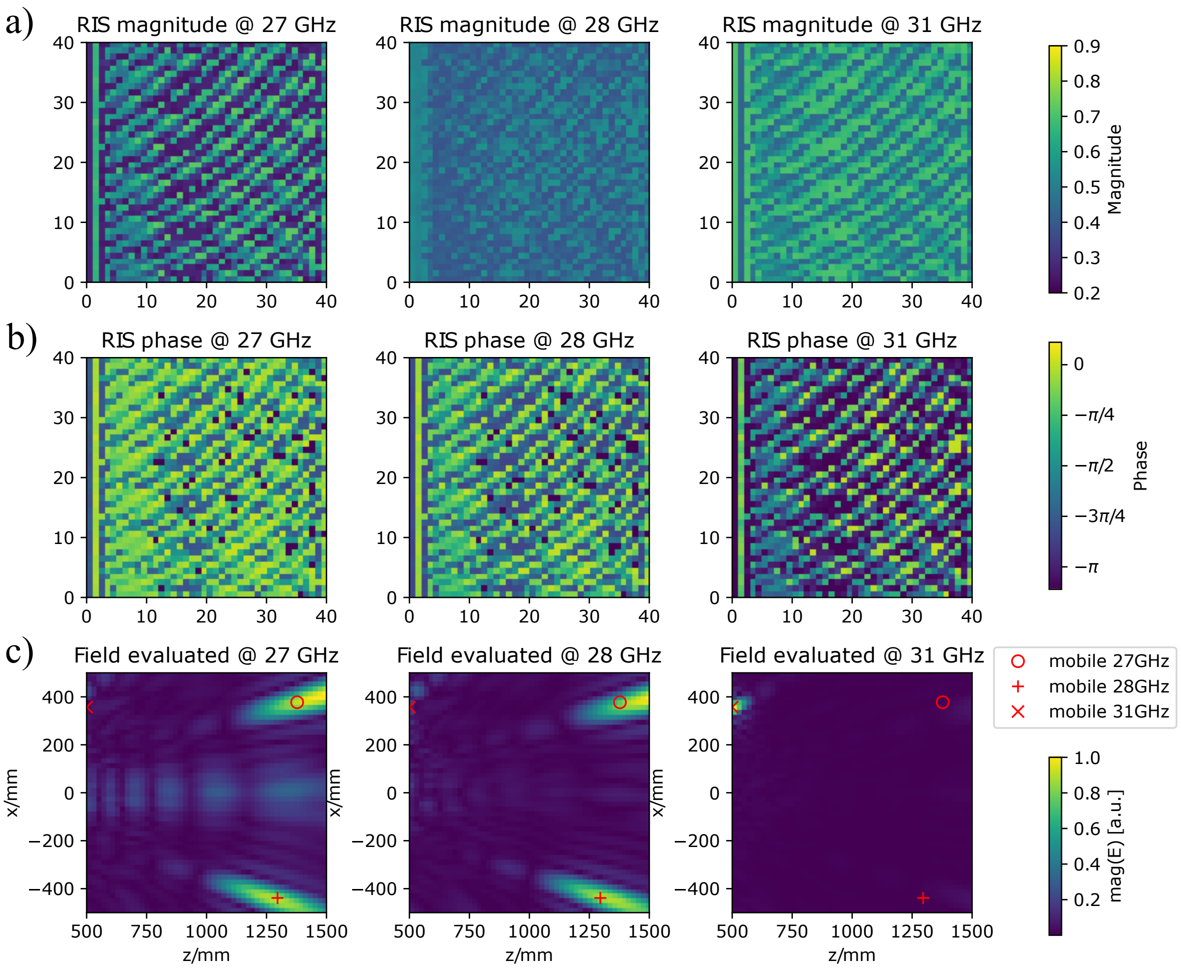

Figure S5. 3rd exemplary configuration of a three-user scenario: The three columns correspond to the three working frequencies at 27 GHz, 28 GHz and 31 GHz. (a) magnitudes of the reflection coefficient for the optimized bias voltage, (b) Respective phases of the reflection coefficients, (c) Calculated electric fields in arbitrary units and the target positions of the foci for the three user frequencies. We observe that MU-FSBS can create foci at each of the target positions as expected.
